# *Campylobacter* bacteriophage DA10: an excised temperate bacteriophage targeted by CRISPR-cas

**DOI:** 10.1186/s12864-020-06808-3

**Published:** 2020-06-12

**Authors:** Steven Hooton, Daniela D’Angelantonio, Yang Hu, Phillippa L. Connerton, Giuseppe Aprea, Ian F. Connerton

**Affiliations:** 1 School of Biosciences, Division of Microbiology Brewing and Biotechnology, University of Nottingham, Sutton Bonington Campus, Leicestershire, LE12 5RD UK; 2 Istituto Zooprofilattico Sperimentale dell’Abruzzo e del Molise “G. Caporale”, Teramo, Italy

**Keywords:** Campylobacters, *Campylobacter* bacteriophages, Prophage, Lytic phage, CRISPR-mediated immunity, Evolution

## Abstract

**Background:**

Lytic bacteriophages that infect *Campylobacter* spp. have been utilized to develop therapeutic/decontamination techniques. However, the association of *Campylobacter* spp. and bacteriophages has been the focus of several strands of research aimed at understanding the complex relationships that have developed between predators and prey over evolutionary time. The activities of endogenous temperate bacteriophages have been used to evaluate genomic rearrangements and differential protein expression in host cells, and mechanisms of resistance to bacteriophage infection in campylobacters such as phase variation and CRISPR-mediated immunity.

**Results:**

Temperate bacteriophage DA10 represents a novel excised and infective virus capable of replication in a restricted set of *C. jejuni* and *C. coli* hosts. Whole genome sequencing reveals that DA10 (35,379 bp) forms part of a novel group of temperate bacteriophages that have limited distribution among database host genome sequences. Analysis of potential host genomes reveals a robust response against DA10 and DA10-like bacteriophages is driven by CRISPR-mediated immunity with 75% of DA10 ORFs represented as ~ 30 bp spacer sequences in numerous *Campylobacter* Type II-C CRISPR arrays. Several DA10-like homologues have been identified in a small sub-set of *C. jejuni* and *C. coli* genome sequences (ranging from near complete integrated prophage sequences to fragments recognisable in the sequence read archive).

**Conclusions:**

A complete intact DA10-like prophage in *C. jejuni* CJ677CC520 provides evidence that the associations between host and DA10-like bacteriophages are long-standing in evolutionary timescales. Extensive nucleotide substitution and loss can be observed in the integrated DA10-like prophage of CJ677CC520 compared to other relatives as observed through pairwise genome comparisons. Examining factors that have limited the population expansion of the prophage, while others appear to have thrived and prospered (Mu-like, CJIE-like, and lytic *Campylobacter* bacteriophages) will assist in identifying the underlying evolutionary processes in the natural environment.

## Background

*Campylobacter* spp. are recognized as being a major cause of gastrointestinal disease on a global scale [1]. Genomic data representative of ecologically-diverse and widely-distributed species such as *C. jejuni*, *C. coli,* and to a lesser extent other *Campylobacter* spp. has advanced our understanding of the genetic factors driving the worldwide success of these pathogens [2–4]. As with many bacteria *Campylobacter* spp. are known to harbour prophage/prophage remnants, for example the *C. jejuni* integrative elements – CJIE, and Mu-like bacteriophage (phage) sequences [5–7]. It is widely accepted that lysogenic conversion of a host bacterium following integration of a temperate phage genome can alter the virulence profile of the host [8]. In campylobacters the situation is not so clear. Genomic rearrangements mediated by the activity of Mu-like prophages (CJIE1 also known as CMLP-1/CampMu-like phage 1) are recognized as major factors governing host resistance to phage predation [7]. Furthermore, some CJIE1-like elements are associated with dampening the host’s ability to acquire exogenous DNA via natural transformation through the activity of an extracellular secreted DNase enzyme [9–11]. A study involving *C. jejuni* encoding homologues of CJIE1 elements indicated a potential role in virulence due to enhanced adherence and invasion of INT407 cells compared to *C. jejuni* lacking these integrated elements [12]. However, a comparable study found no correlation between the presence/absence of CJIE1-like elements and the ability of *C. jejuni* to adhere to and invade HT29 colon cancer cells [13]. A further set of integrated genetic elements (CJIE2, CJIE3, and CJIE4) can also be found embedded in many *C. jejuni* genomes. Whilst CJIE2 and CJIE4 possess hallmark features of prophage/prophage remnants, CJIE3 most likely arose following integration of a plasmid-like mobile element into the host chromosome [14]. Interactions between *Campylobacter*-associated mobile genetic elements can also be observed in the large multidrug resistance plasmid of *C. jejuni* T1–21 [15]. Intriguingly, the 82.7 Kb mega-plasmid contains a 45 Kb Mu-like prophage region that may be a remnant of a transposition event outside of the established lytic and lysogenic lifecycles. The general consensus is that a non-inducible or as yet unidentified mechanism is employed for the activation of CJIE1 and CJIE-like elements [16], although recourse to mitomycin-c has been reported to induce CJIE1 [12, 14]. It has also been shown that CJIE1 and other relatives (CJIE4) have the potential to influence protein expression of their bacterial lysogens [16].

In comparison to other enteric pathogens such as *Escherichia coli* and *Salmonella* spp. [17] there is a distinct lack of temperate phages known to infect, undergo chromosomal integration, and subsequently excise to produce nascent infectious virions in *C. jejuni* and related species. However, there are historical reports of the spontaneous induction of temperate phages harboured by *C. fetus* (formerly described as *Vibrio fetus*) [18, 19]. Transmission electron micrographs obtained of phages targeting *C. fetus* indicate a *Siphoviridae* morphology as indicated by the presence of long, non-contractile tail machinery [19, 20]. Multiple mechanisms exist for bacteria to resist phage infection, such as phase variation of cognate receptors, switching from motile to sessile modes of growth, and CRISPR systems. It has been established that *Campylobacter* spp. employ several mechanisms to evade phage, including genetic rearrangement [7], phase variation [21], the use of an alternative flagellin [22], and the acquisition of CRISPR spacers to abrogate phage infection [23, 24]. Alternatively, phages have the capacity to mutate and overcome modifications such as phase-varied cognate receptors, leading to a classical co-evolutionary arms race. Class II/III *Campylobacter* phages (*Eucampyvirinae* Firehammervirus and Fletchervirus) are capable of interfering with host CRISPR systems via interactions between phage-encoded Cas4 protein and the host Type II-C system [23, 25].

*Campylobacter* phage DA10 was isolated from poultry-associated samples during a study performed in 2017 [26]. Analysis of the lytic properties of DA10 indicate a limited host-range against a diverse panel of *Campylobacter* spp. (7/118). Morphologically DA10 has been reported to display classic *Myoviridae* features including a icosahedral head, neck-like structure and a contractile tail [26]. However, here we report DA10 has a novel small genome compared to virulent *Campylobacter* bacteriophages that is related to prophage in the growing number of *Campylobacter* genome sequences available in the database. Despite a distinct lack of inducible prophages in *Campylobacter* genomes, DA10 represents a rare excised and infective phage arising from biological interactions between campylobacters and associated phages. However, extensive representation of DA10 sequences in the CRISPR arrays of diverse campylobacters likely accounts for the restricted host and the progression towards extinction of the infectious virus. Moreover, the degradation of prophage sequences in *Campylobacter* genomes exemplifies the evolutionary pressures exerted by the *Campylobacter* Type II-C CRISPR-Cas system.

## Results

### Characterisation of bacteriophage DA10

**Fig. 1:**
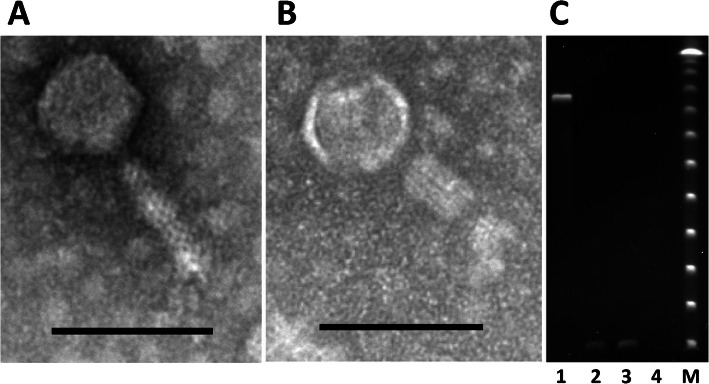
TEMs and PFGE of *Campylobacter* bacteriophage DA10. Panel **a** shows a transmission electron micrograph image of phage DA10. The bar represents 100 nm. Panel **b** shows a transmission electron micrograph image of phage DA10 with a contracted tail. The bar represents 100 nm. Panel **c** shows pulse fields gel electrophoresis of bacteriophage genomic DNAs: lane 1, Class I *Campylobacter* phage 12 DNA; lane 2, Phage DA10 DNA; lane 3, Phage DA10 DNA digested with RNase A; lane 4, Phage DNA DA10 digested with DNase 1; lane M, concatenated lambda phage DNA (48.5 kb) size marker

Transmission electron microscopy confirmed the morphology of phage DA10 to feature a contractile tail with an icosahedral head (Fig. 1a and b) indicating phage DA10 is a myovirus. The phage tails had a mean length of 93.5 ± 3.7 nm and width of 21.6 ± 3.0 nm. The phage tails were shorter than previous observations of virulent class II/III *Campylobacter* phages (115–148 nm) [27, 28]. The mean head diameter of 67 ± 3.7 nm was also reduced compared to the class II/III *Campylobacter* phages (92–96 nm), suggesting DA10 represents a new class of *Campylobacte*r phage. Figure 1c shows PFGE of the D10 genome, which indicates the genome size to be ≤48.5 kb that represents the lowest molecular weight marker present. This is in contrast to the class I *Campylobacter* bacteriophage 12 DNA on the same gel that is estimated to be 420 kb. The DA10 PFGE band is resistant to RNase digestion but sensitive to DNase1, indicating the phage has a DNA genome (Fig. 1c). Bacteriophage DA10 therefore represents the smallest *Campylobacter* phage DNA genome recorded, which prompted investigation of the genome sequence.

### Genome features

**Fig. 2:**
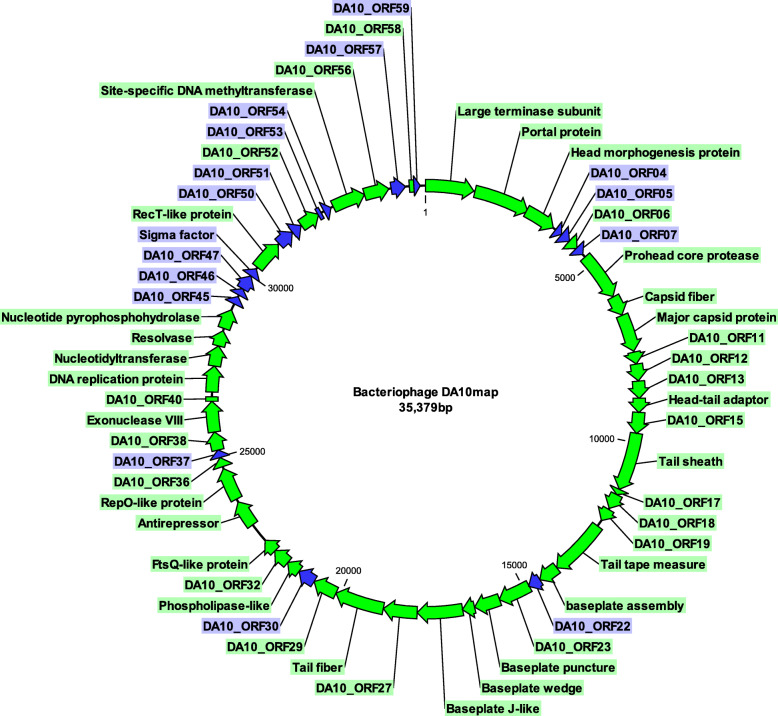
Circular map of *Campylobacter* bacteriophage DA10. Open reading frames are identified by number or the putative protein they encode. Green arrows represent ORFs for which CRISPR spacers have been identified in *C. jejuni*, *C. coli*, and *C. hyointestinalis*. Blue arrows identify ORFs that are absent from *Campylobacter* CRISPR arrays

To further our understanding of phage DA10 whole genome sequencing was performed. Assembly of DA10 sequence reads produced a complete circularly permuted genome of 35,379 bp (Fig. 2) with 600-fold coverage. Many temperate bacteriophages have discrete ends, which prompted us to examine the accumulation of sequence reads that might implicate the presence of terminal repeats. No regions of read accumulation were observed, although these may not be evident due to the tagmentation protocol adopted for DNA sequence library preparation. The genome sequence was therefore deposited in the nucleotide sequence database as a putative circularly permuted genome (Acc. No. MN530981). Analysis of the nucleotide composition of DA10 indicates a GC content of 27.1% which is slightly below that of *C. jejuni* (~ 30% GC). BLASTn analysis of the DA10 nucleotide sequence identified a near intact relative of DA10 present as an integrated prophage in *C. jejuni* CJ677CC520 [29]. CJ677CC520 (Acc. No. CP010501) was isolated from human faeces/blood and is a member of sequence type 677 (ST677). The predicted size of the CJ677CC520 prophage is 36,401 bp (base position 446,910-483,310) and is integrated downstream of a tRNA 2-selenouridine gene. Approximately 20.2 Kb of the phage DA10 genome can be mapped to the prophage region in CJ677CC520 with nucleotide identities ranging from 72 to 96%. Several contigs (5–37 Kb) recently uploaded as part of a study into *Campylobacter* antimicrobial resistance show that DA10-like prophages are present in a small number of *C. jejuni* and *C. coli* chromosomes (using DA10-like anti-repressor protein as an identifier). Of these, six complete circularly permuted DA10-like genomes have been identified (Table 1 shows a pairwise comparison of the nucleotide identities and Table 2 post Mummer alignment of the coding regions). Hallmark features of DA10-like phages include genome sizes ranging from ~ 33 to 38 Kb in length, a conserved gene set encoding major structural proteins, DNA replication/repair/recombination functions, and DNA methylation. Similar %GC contents are also observed for DA10-like phages with ranges between 26.2% (as observed for the integrated prophage in CJ677CC520) and 27.8% (putative prophage in *C. coli* NC_C4236).

**Table 1:** Pairwise comparison of nucleotide identities for DA10 and DA10-like sequences

	DA10	PNUSA C003578	CcNC_C4236	CcNC_C4247	CjFSIS 11,811,828	PNUSA C003324	CJ677 CC520
**DA10**	*	24,042/ 35,379	23,679/ 35,379	24,064/ 35,379	23,170/ 35,379	26,403/ 35,379	18,052/ 35,379
**PNUSA** **C003578**	23,605/ 37,072	*	20,967/ 37,072	20,967/ 37,072	21,273/ 37,072	29,909/ 37,072	19,925/ 37,072
**CcNC_C4236**	23,520/ 33,831	22,435/ 33,831	*	29,470/ 33,831	22,986/ 33,831	23,927/ 33,831	17,738/ 33,831
**CcNC_C4247**	20,413/ 33,823	20,262/ 33,823	27,669/ 33,823	*	17,782/ 33,823	21,231/ 33,823	14,885/ 33,823
**CjFSIS** **11,811,828**	23,899/ 33,563	18,783/ 33,563	19,295/ 33,563	19,097/ 33,563	*	22,012/ 33,563	18,704/ 33,563
**PNUSA** **C003324**	26,142/ 37,779	27,760/ 37,779	21,504/ 37,779	21,504/ 37,779	22,140/ 37,779	*	19,988/ 37,779
**CJ677** **CC520**	16,146/ 37,405	16,785/ 37,405	14,016/ 37,405	14,217/ 37,405	15,403/ 37,405	16,032/ 37,405	*

**Table 2:** Pairwise comparison of nucleotide identities for DA10 and DA10-like sequences post Mummer alignment of the coding regions

	DA10	PNUSA C003578	CcNC_C4236	CcNC_C4247	CjFSIS 11,811,828	PNUSA C003324	CJ677 CC520
**DA10**	*	28,824/ 35,379	25,789/ 35,379	25,791/ 35,379	25,635/ 35,379	31,207/ 35,379	10,263/ 35,379
**PNUSA** **C003578**	28,932/ 37,072	*	24,273/ 37,072	24,275/ 37,072	23,575/ 37,072	34,654/ 37,072	10,299/ 37,072
**CcNC_C4236**	25,858/ 33,831	24,284/ 33,831	*	33,616/ 33,831	20,797/ 33,831	26,190/ 33,831	8529/ 33,831
**CcNC_C4247**	26,066/ 33,823	24,493/ 33,823	33,823/ 33,823	*	21,006/ 33,823	26,405/ 33,823	8536/ 33,823
**CjFSIS** **11,811,828**	25,683/ 33,563	23,494/ 33,563	20,738/ 33,563	20,740/ 33,563	*	24,894/ 33,563	10,308/ 33,563
**PNUSA** **C003324**	31,020/ 37,779	34,629/ 37,779	25,651/ 37,779	25,659/ 37,779	24,880/ 37,779	*	11,401/ 37,779
**CJ677** **CC520**	10,327/ 37,405	10,256/ 37,405	8184/ 37,405	8191/ 37,405	10,399/ 37,405	11,396/ 37,405	*

Although no genes encoding an integrase could be identified in DA10 or DA10-like DNA, a putative Holliday junction resolvase (DA10_ORF43) was identified at 20% amino acid identity to bacteriophage T7 endonuclease 1 (HHpred *p* > 99%, expect = 1.7e-12). Small fragments of the DA10 genome are found in *Campylobacter* spp. chromosomes (*C. jejuni*/*C. coli*), Class II/III *Campylobacter* phage genomes, and CJIE prophage sequences. DA10 and DA10-like phages therefore constitute a unique mosaic genome structure displaying weak, but recognizable associations with *Campylobacter* prophages, prophage remnants, and lytic *Campylobacter* phages. Complete or extended DA10-like prophage sequences are rare when surveying whole genome studies lodged in the NCBI Sequence Read Archive (10 of 29,736 genomes for *C. jejuni* and 12 of 11,916 genomes for *C. coli*), suggesting that DA10 has a limited ecological distribution in *Campylobacter* populations. The exception to this is a 1.5 Kb region of the DA10 genome (bases 21,460-22,990) that is conserved across multiple *Campylobacter* genomes. In DA10 this region contains genes encoding hypothetical proteins and a FtsQ related protein (HHpred *p* > 96%, expect = 0.015). Internal to this is a 470 bp region conserved in CJIE4 prophage sequences (ANI of 93%): CJIE4–2 (KF751794), CJIE4–3 (KF751795), CJIE4–4 (KF751797) (436/470 bases), CJIE4–1 (KF751793) (391/425 bases) and CJIE4–5 (KF751797A) (394/425 bases). Frequently two copies are present at different genomic locations in *Campylobacter* genome sequences. For example, in *C. jejuni* FDAARGOS_421 (CP023866) this sequence is encoded at ~ 0.87 Mb and ~ 1.69 Mb in the host chromosome.

Examination of the DA10 genome identified a total of 59 open reading frames (ORFs) encoding 44 hypothetical proteins, and 15 ORFs that could be assigned putative functions based on BLASTp analysis of translated amino acid sequences (Fig. 2). All 59 DA10 ORFs are found on the same strand with no putative genes being identified on the opposite strand. For genes that could be assigned putative functions several were associated with the synthesis of phage structural proteins (tail-associated proteins, baseplate assembly, major capsid/head morphogenesis), nucleotide metabolism (sugar phosphate nucleotidyltransferase), and DNA replication/regulation (site-specific methyltransferase, RecT-like, RepO-like, and phage antirepressor). Many of the proteins encoded by DA10 have sequence similarities to a small subset of *C. jejuni* and *C. coli* genome sequences further hinting towards a limited distribution of this phage throughout *Campylobacter* populations.

### Orthologous relationships

Opening the DA10 genome sequence at the recognisable large terminase subunit gene encoded by DA10_ORF1 (located at nucleotide positions 1–1284 in Fig. 2). BLASTn analysis indicates the gene encoded by DA10 displays 86% ANI (1087/1264) when aligned against its homologue in CJ677CC520. Several *C. jejuni* and *C. coli* genomes are observed to encode similar proteins ranging from 83 to 99% identity at the amino acid level, with some examples showing two separate proteins separated by an internal stop codon (positions 1–248 and 248–427). Similar large terminase subunits were identified in *Helicobacter* sp. (162/432 amino acid identity) and *Helicobacter aurati* (167/427 amino acid identity). *Helicobacter*-encoded large terminase subunits contain conserved protein domains associated with the packaging of phage DNA during the virion assembly process. While these domains were not present in the DA10 large terminase protein sequence, numerous alignments to residues associated with Terminase_3 (pf04466) and Terminase_6c RNase H-like (pf17289) domains were identified.

DA10_ORF10 encodes the putative major capsid protein in the DA10 genome (nucleotides 6575-7570). BLASTn analysis of the gene sequence indicates 673/994 (71% ANI) with its homologue encoded in CJ677CC520. Several protein homologues are observed when the translated 331 amino acid sequence is aligned with conserved hypothetical proteins encoded in *C. jejuni* and *C. coli* genomes. Limited homology of the DA10 amino acid sequence is evident when aligned with major capsid proteins of various phages. For example, *Pseudomonas* phages NP3 (AMQ76141), PA5 (APD20704), and KPP22 (BAU20690) all display identical alignments over 28% of the DA10 sequence. Interestingly, the majority of phage-associated BLASTp hits with DA10 major capsid protein are found to be with highly divergent homologues found in *Pseudomonas* phages. No obvious homology at the amino acid level was observed with the major capsid proteins present in Group II/III *Campylobacter* phages or CJIE-like elements.

Recognisable ORFs encoding a putative phage tail fiber protein (DA10_ORF28; bases 18,813 to 20,126), tail sheath protein (DA10_ORF16; bases 9749 to 11,296) phage-associated DUF2612 domain-containing protein (DA10_ORF27; bases 17,914 to 18,810), and a baseplate J-like protein (DA10_ORF26; bases 16,709 to 17,914) are present in the DA10 genome. A small-subset of *C. jejuni* and *C. coli* possess near-identical homologues of the 272 amino acid phage-associated protein encoded by DA10_ORF28, and weaker alignments are observed with proteins encoded in *C. lari* and *C. ornithocola*. Amino acids 89–176 display similarity (~ 36% identity) to a hypothetical protein encoded by several Group III *Campylobacter* phages, and Group II phage CP21. BLASTp hits for short regions of the putative tail fiber sequences from phages infecting *Pectobacterium* spp. and *Erwinia amylovora*. DA10_ORF27 encodes a 298 amino acid protein containing a DUF2612 domain-that is conserved in several *C. jejuni* and *C. coli* genomes, as well as multiple highly diverged homologues in numerous bacterial species. DUF2612-domains (pfam11041) are widely distributed in phages/prophages associated with *Proteobacteria*. DA10_ORF16 encodes a protein of 515 amino acids that is predicted to share structural similarity to a R-type phage tail-like bacteriocin contractile sheath (HHpred *p* > 99%, expect = 7.6e-20) that forms a cell penetrative structure with a baseplate, receptor-binding tail fibers and an inner needle-like tube analogous to bacteriophage infection machinery. DA10_ORF26 encodes a 401 amino acid baseplate J-like protein including the conserved baseplate J family domain (pfam04865) indicating that this ORF encodes an important structural component of the DA10 phage particle. Alignment of the protein sequence with the baseplate J-like protein encoded by CJ677CC520 indicates 251/396 (63%) identity. Sequential nucleotide database searches of the tail fiber region revealed short dispersed regions of similarity, including a 530 bp region (bases 18,425-18,955) (375/530–70% average nucleotide identities - ANI); 131 bp (95/131–73%) and 72 bp regions (54/72–75%) that align to the integrated prophage encoded in CJ677CC520. A further 92 bp region is highly similar to Class II *Campylobacter* phage CP21 (74/92–80%). Adjacent to the baseplate J-like encoding gene are predicted ORFs of related function, notably encoding baseplate wedge (DA10_ORF25), puncture (DA10_ORF24) and assembly proteins (DA10_ORF21).

DA10_ORF42 encodes a 543 bp sugar phosphate nucleotidyltransferase gene (bases 27,415 to 27,957). Conserved homologues (76% ANI) of DA10_ORF42 are found to be encoded in Class III *Campylobacter* phage genomes (CP81, CP39, CP8, CPX, CP81, PC5, CP30A, PC14, NCTC 12673, and vB_CjeM_Los1). *C. coli* OR12 and an aerotolerant derivative (CP019977) contain two homologues of the 543 bp sugar phosphate nucleotidyltransferase gene encoded by DA10 [30]. Both genes in *C. coli* OR12 display 79% ANI (399/508) over a 508 bp region of the DA10 sequence. The first copy is located at ~ 1.76 Mb and the second copy at ~ 1.9 Mb on the *C. coli* OR12 genome. Duplication of this gene in *C. coli* OR12 is interesting as while several other *C. coli* genomes contain an identical gene, OR12 is the only example observed to possess two copies. Examination of the regions within *C. coli* OR12 highlights that both copies are encoded within intact prophage sequences present in the host genome. However, these integrated prophages in *C. coli* OR12 display very little homology towards DA10-like phage sequences. *C. coli* OR12 possesses another duplication of a gene encoded by DA10 (DA10_ORF43). This ORF is predicted to encode a resolvase similar to enterobacteria phage T7 that is not present in other *C. coli* sequences. The sequence of DA10 aligns with 87% ANI (214/246) against two copies of this gene in the *C. coli* OR12 genome at ~ 1.25 and ~ 1.42 Mb [30].

DA10_ORF44 (bases 28,439-28,969) was identified as a nucleotide pyrophosphohydrolase (HHpred *p* > 99%, expect = 6.1e-10), which displays sequence identity to Class III *Campylobacter* phage DNA sequences: PC5 (KX229736), CP81 (NC_042112), CP8 (KF148616), CPX (JN132397), CP30A (JX569801), NCTC 12673 (GU296433), PC14 (KX23633), and vB_CjeM_Los1 (NC_041896). Multiple alignments of the amino acid sequence to a conserved hypothetical protein present in *C. jejuni*/*C. coli* genomes indicate that this phage-associated protein may have prophage associations. The ORF encoded in DA10 encodes 5 bp repeats of 5′-AAAAA-3′ at each end suggesting a possible recombination event has allowed for horizontal transfer of this gene.

A putative site-specific DNA methyltransferase is encoded in DA10_ORF55 (bases 32,866-33,750). Translation of the gene sequence produces a 294 amino acid protein homologous to site-specific DNA methyltransferases encoded in three *C. jejuni* and four *C. coli* genomes. No other matches with *Campylobacter* spp. or *Campylobacter* phage-associated proteins were identified. Alignments (43% identity) with prophage-encoded site-specific (N6_N4) methyltransferases from *Streptococcus mutans* (WP_002287175), *S. mutans* NLML9 (EMC100053), and *S. mutans* 24 (EMC47101) signify a possible function for the DA10-encoded protein. However, no conserved protein domains associated with N6_N4 DNA methylation or similar were identified in the DA10 site-specific DNA methyltransferase. An interesting match is also observed with a putative DNA methylase in *Lactobacillus* phage LBR48 (ADF83450) with 127/282 (45% identity).

### DNA regulation

DA10_ORF41 (bases 26,726-27,418) encodes a 230 amino acid AAA-family ATPase protein with homologues observed in *C. jejuni*, *C. coli*, *C. fetus*, and *C. iguaniorum*. BLASTn analysis of the gene sequence indicates that it has very limited distribution in sequences deposited in the database. However, when aligned to its counterpart encoded in CJ677CC520, a high degree of conservation 94% ANI (652/693) is observed. Translated protein alignments with *C. jejuni* and *C. coli* proteins show a higher degree of conservation (72–96% identities) than those observed for *C. fetus* and *C. iguaniorum* (41%). Analysis of conserved protein domains (P-Loop NTPase superfamily [cl21455] AAA-family of ATPases) indicate a potential functional role in the replication of DA10 DNA. A single match of limited homology (22% amino acid identity) is evident with a DnaC-like protein encoded in *Streptococcus* phage 73 (KT717083). No alignments with any other phage-encoded proteins are observed at the nucleotide or protein level.

A putative exonuclease VIII-like protein is encoded by DA10_ORF39 (816 bp, bases 25,668-26,483). At the nucleotide level CJ677CC520 is most similar with 93% ANI (760/816) aligning with DA10_ORF39. The 271 amino acid exonuclease VIII-like protein has several near-identical homologues (93–99%) encoded by *C. jejuni* and *C. coli* present in the database. A conserved protein domain (PRK09709) indicates the exonuclease VIII-like function of this protein, and numerous weak alignments are observed with similarly annotated proteins in diverse genomes such as *Synechococcus* phage S-CBS1 (ADP06638.1) and Cyanophage KBS-S-2A (AGH57650.1).

Analysis of the putative antirepressor gene encoded by DA10_ORF34 (bases 22,997-23,713) indicates potential roles in DNA binding and regulation. DA10 antirepressor is highly homologous to antirepressor/BRO family proteins found in *C. jejuni* and *C. coli* (77–88% amino acid identity). Although the DA10 antirepressor lacks some of the canonical conserved KilAC-domain residues (pfam03374) that are often associated with antirepressor proteins, several amino acids are conserved. Immediately downstream of DA10 antirepressor is DA10_ORF35 (bases 23,798-24,673) that encodes a putative 291 amino acid RepO-like protein. Two single examples of homologues of DA10 RepO-like protein are observed in *C. jejuni* (EAL7532945) and *C. coli* (EAH9758457). Analysis of the amino acid sequence of DA10 RepO-like protein indicates that it is modular in nature with distinctive N- and C-terminal domains. Linking these two protein domains is a sequence motif of 16 amino acids. Residues 165–291 show conservation with several conserved hypothetical proteins in *C. jejuni* and *C. coli*. However, the N-terminal residues (1–164) are distinctly different from that observed in phage DA10. A similar situation is evident when aligning the RepO-like protein of DA10 against the CJ677CC520 prophage. No N-terminal similarity can be observed between the two RepO-like proteins, whereas the C-terminus exhibits 99% identity between amino acid residues 160–291. At the nucleotide level it is apparent that the linker region of DA10_ORF35 consists of three short polyA tracts (AAAAAAATATAAAAAAAGAAAAAAAA) that may be capable of promoting recombination thus leading to the alternative N-terminal protein sequences observed.

### *Campylobacter* CRISPR spacers targeting DA10

Small fragments of the DA10 genome can be identified as spacer sequences in *Campylobacter* CRISPR arrays (Table 3) that target 43 of the 59 ORFs of DA10 (Fig. 2). The DNA sequences are present as 29–38 bp CRISPR spacers in the Type II-C systems encoded by campylobacters. A spacer targeting DA10_ORF13 appears more frequently than any other DA10-associated CRISPR spacers identified during this analysis (5′-AACCCCGCTAACTGCATAAAAATATCCATTTTC-3′). Importantly it appears that a considerable number of DA10 genes can be identified as ~ 30 bp spacers in *Campylobacter* CRISPR arrays, suggesting a robust and widespread defence system is being deployed in campylobacters to mitigate DA10-like phage infection. In some cases multiple spacer sequences targeting DA10 are observed within the same CRISPR array. For instance, *C. jejuni* R123 (HQ378289) has acquired the widely distributed spacer sequence described above as well as spacers targeting the tail tape measure and baseplate J-like genes. Some spacers are observed to have a single base mismatch due to the presence of an extra nucleotide in the DA10 gene relative to the CRISPR sequence (e.g. cytosine insertion in the *C. jejuni* HCC91 spacer targeting RecT-like recombination/repair gene). Many other 30–35 bp alignments originating from the DA10 genome are present in a wide range of *Campylobacter* genomes, presumably these short alignments map to unannotated CRISPR sequences. It is interesting to note that the genome sequence of CJ677CC520 containing the DA10-like prophage element has an atypical Type II-C CRISPR system configuration with Cas2 and Cas9 present, and Cas1 notably absent (nucleotide positions 1,419,058-1,422,456). The DNA endonuclease function associated with cas1 is critical to the CRISPR adaptation process. Spacer acquisition and adaptation in CRISPR systems arises from Cas1-Cas2 complexes that generate protospacers from foreign DNA [31]. It is possible that a malfunctioning CRISPR system has allowed DA10-like phage infection of CJ677CC520 to establish a lysogenic state. Several examples also exist of CRISPR spacers targeting the DA10 antirepressor gene sequences.

### Non-CRISPR targeted DA10 ORFs

Sixteen ORFs in the DA10 genome are not represented as spacers in *Campylobacter* CRISPR arrays. Analysis of these ORFs suggests that the majority of these are likely derived from host DNA as reflected by matches with hypothetical proteins encoded in a number of *C. jejuni* and *C. coli* genomes. Notably DA10_ORF4, ORF5, ORF7, ORF25, ORF22, ORF45, ORF46, ORF47, and ORF48 appear to be conserved hypothetical proteins in this category. DA10_ORF53 is a 96 bp gene that displays homology to a small fragment encoding a hypothetical protein in *Campylobacter* pVir-like plasmids. For DA10_ORF50, no significant BLASTn matches were identified in genomes of *Campylobacter* spp. or associated phages. Translation of the nucleotide sequence and subsequent BLASTp analysis identified a single example of a protein homologue (EYI48_08780) present in a 9471 bp contig originating from a sequence read archive (SRR8592636) of a *C. coli* cattle isolate. Conserved proteins that are found in DA10 and other *Campylobacter* phage genomes also appear to be free from CRISPR-mediated immunity. In this context DA10_ORF30, ORF37, ORF54, and ORF57 all display conservation with *Campylobacter* phage-associated proteins. DA10_ORF57 (384 bp) encodes a 127 amino acid protein that is conserved in *Campylobacter* phages CP20, CPt10, CP81, CP8, CPX, CP81, CP30A, PC14, NCTC 12673, PC5, CP39, CP21, and vB_CjeM_Los1. A similar observation is made with DA10_ORF54 with similar proteins encoded in CPt10, CP20, CP39, CP8, CPX, CP81, and vB_CjeM_Los1. A further match against *Campylobacter* phages CP39 and PC5 can be found when analysing DA10_ORF37 (207 bp), which encodes a small 68 amino acid protein. DA10_ORF30 (474 bp) encodes a 157 amino acid protein that is homologous to counterparts present in *Campylobacter* prophages CJIE4–2 and CJIE4–5.

## Discussion

Phage DA10 and DA10-like phages are present in a limited number of genome sequences associated with their campylobacter hosts. DA10 has been isolated from the environment, has been identified as a *Myoviridae* following TEM analysis, can produce infectious virions following replication in a permissive host, and potentially has a long-standing evolutionary relationship with *Campylobacter* spp. Given the large number of *Campylobacter* spp. genome sequences that have been produced in recent times, and the paucity of DA10-like phage sequences associated with them, it is reasonable to assume that DA10-like phages are rare in nature. DA10-like phages may constitute a novel genus of temperate phages, albeit with extremely limited environmental distribution. Genome sizes ranging from ~ 33–38 Kb are observed in DA10-like phages with GC contents of 27.1–27.2% for those present in *C. jejuni* whilst for *C. coli* a slightly higher value of 27.7–27.8% is observed. The lowest GC content of 26.3% is found in the integrated prophage of CJ677CC520. It is interesting to note that previous studies of CJ677 strains show that this sequence type is associated with a higher degree of disease-severity [32]. Increased resistance to normal human serum was reported for CJ677 bacteraemia-associated strains isolated in Finland from 1998 to 2007 [33]. Infection with CJ677 also typically resulted in longer recovery periods following hospitalization [34].

Identification of a small number of DA10-like phages allowed for a limited comparative analysis to be performed. Table 1 (whole genome alignments) and Table  2 (mummer alignments of coding regions) show the results obtained from nucleotide sequences of all DA10-like phages. Pairwise results from whole genome alignments show that DA10 is most similar at the nucleotide level to *C. jejuni* PNUSAC003324 (74.6% ANI), and alignments of the coding regions show comparable results (88.2% ANI). The integrated DA10-like prophage in CJ677CC520 has suffered considerable degradation in respect of nucleotide substitution and loss in comparison with other members of the group. Pairwise alignment of DA10 with CJ677CC520 prophage results in an overall ANI of 51%, however mummer alignment of the intact coding regions shows a higher degree of sequence divergence (29% ANI), implying preservation of gene function is not essential.

**Fig. 3:**
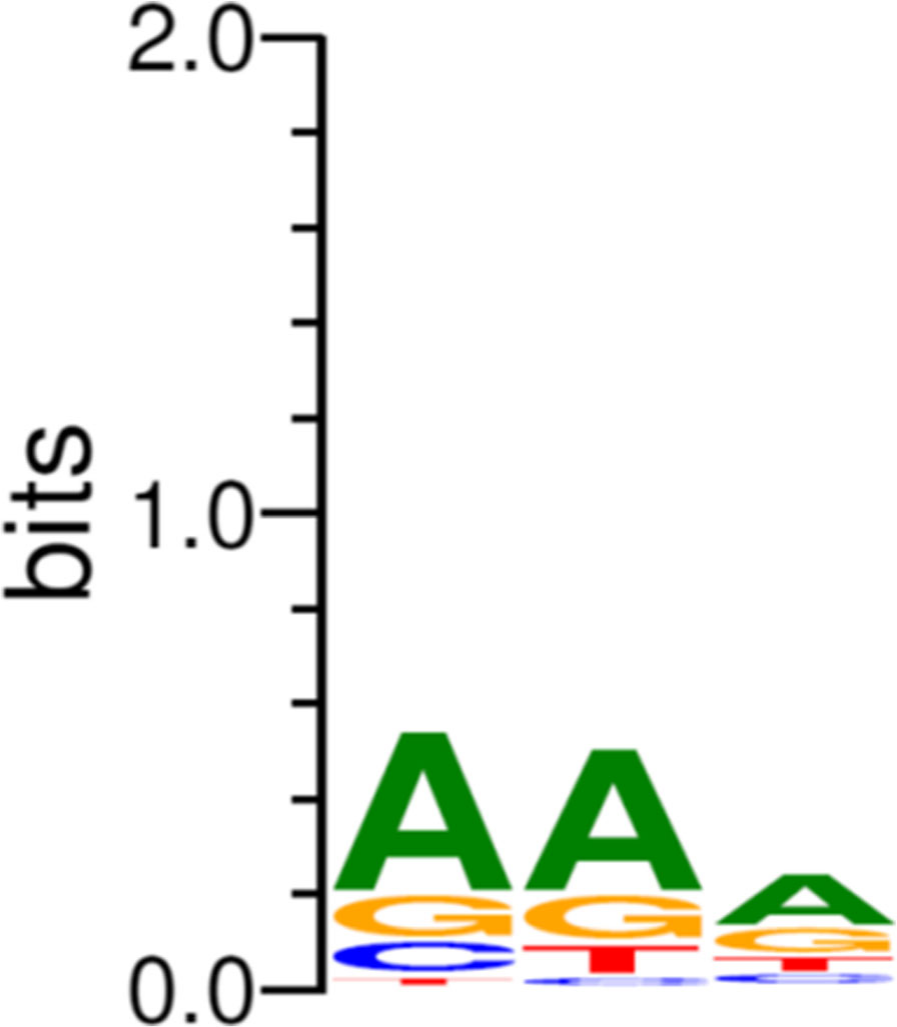
Alignment and frequency of DA10 protospacer adjacent motifs (PAM)

It is quite striking that BLASTn analysis of multiple genes encoded by DA10 delivers search results of ~ 30 bp fragments that are embedded in CRISPR arrays of *C. jejuni* and *C. coli*. Of the 59 ORFs annotated in the DA10 genome, CRISPR spacers targeting ~ 75% of these (43 ORFs) were identified (Fig. 1 and Table 3). Several *C. jejuni* and *C. coli* CRISPR arrays also harbour multiple spacers that are homologous to DA10 DNA. *C. jejuni* YH003 encodes two 30 bp CRISPR spacers that represent perfect matches to DA10_ORF16 and DA10_ORF52. Similarly, *C. coli* BP3183 encodes two identical CRISPR spacers that are homologous to DA10_ORF5 (30/30) and DA10_ORF28 (32/32). *C. coli* BP3183 possesses a second CRISPR spacer targeting DA10_ORF28, which exhibits a single base pair mismatch with its cognate 30 bp sequence in DA10. Other examples are also observed where a CRISPR array harbours two spacers that target DA10 DNA with the caveat that one of the spacers displays multiple mismatches to the target sequence. For *C. jejuni* GB1 this is found within a spacer that targets DA10_ORF4 (37/40 matches), however a second spacer that is 100% identical to DA10_ORF52 (32/32 matches) is also present within the host CRISPR array. Multiple CRISPR spacers targeting the genes encoded of a single phage suggests the phage previously had replicative success but has become an active target. There is a bias towards adenine bases in the protospacer adjacent motifs (PAMs) that arise from DA10, which may contribute to the frequency of targeting the low GC contents of DA10 and DA10-related sequences (Fig. 3). The limited distribution of DA10-like integrated prophages in *C. jejuni* and *C. coli* genomes is testament to a robust CRISPR-mediated defence that targets multiple functional genes in the phage. Circumvention of immunity will be greatly diminished in this scenario as the invading phage genome is unlikely to be able to tolerate the multiple point mutations required to evade CRISPR-mediated responses. In *Pseudomonas aeruginosa* it has been shown that multiple CRISPR spacers targeting a single phage can effectively drive the virion to extinction [35, 36].

**Table 3:** CRISPR spacers targeting *Campylobacter* phage DA10

Host (Acc. No.)	CRISPR spacer sequence (ID)	DA10 target
*C. coli* BP3183 (CP017871)	CACGTGTGGGAAGGAGAACCTTTAGAGTATAA (32/32)	DA10_ORF01
*C. jejuni* HSA43 (KR155141)	CCTTTGTCAATTTATGAAAAATTATGTTTCAA (32/32)	DA10_ORF02
*C. jejuni* HCC31 (KR155133)	AAATTTACAGCACAAAATAAAGATTTTAATATT (33/33)	DA10_ORF03
–	No matches	DA10_ORF04
–	No matches	DA10_ORF05
*C. jejuni* F041 (EF017333)	TAGATTTAGAAACTTTTAATAAAGTAAAAGA (31/31)	DA10_ORF06
–	No matches	DA10_ORF07
*C. jejuni* R17 (HQ378296)	ATAATCCTAGCGAGGAAAGCAAAACTGATGATG (33/33)	DA10_ORF08
*C. coli* YH503 (CP025281)	TGGTTGCTTCGTTCAATCAAAAACAGGTGC (30/30)	DA10_ORF09
*C. jejuni* NCTC 13268 (LR134497)	AGTGCTACAGCCTTTACCACACTTCAATC (29/29)	DA10_ORF10
*C. hyointestinalis* LMG9260 (CP015575)	TTCAAAAGATGA-TTTTGATTTACTTTGCAAATCTT (32/36)	DA10_ORF11
*C. coli* RM4661 (CP007181)	ACCCTGAGTTTAAAGACTTAAGTAAATGTAA (31/31)	DA10_ORF12
*C. jejuni* YH003 (CP041584)	AGGGGTGATATTGTTATGAGTATTATGCAA (30/30)	DA10_ORF13
*C. jejuni* F087 (EF017335)	TTAATCCTAGTGAAATTGCAAAATTAACAAG (31/31)	DA10_ORF14
*C. jejuni* HCC01 (KR155140)	GTTAAAATCTTTCGAAACAATAGAATATTTA (30/31)	DA10_ORF15
*C. jejuni* HCC92 (KR155175)	GAAAATGGATATTTTTATGCAGTTAGCGGGGTT (33/33)	DA10_ORF16
*C. jejuni* HSA25 (KR155156)	GTGGCTAATAATAATAAAGCTAAGACTGATA (31/31)	DA10_ORF17
*C. coli* 210 (KR155130)	TGTAGCTGGTAAGGTTATAAGAGGTTTATT (30/30)	DA10_ORF18
*C. jejuni* ATCC 33560 (CP019838)	TTAGTGCTTTATTTGAAAATGAGATGGCAG (30/30)	DA10_ORF19
*C. coli* RM4661 (CP007181)	TTTTCTTATGAAGTTTTTAAAGGAATTATAC (31/31) GCTTTAGGAAATGCTTTAAAACGCTTTGG (29/29)	DA10_ORF20
*C. jejuni* HCC01 (KR155140)	TATAAAAATATGAAAATAGTATCTTACGCA (30/30)	DA10_ORF21
–	No matches	DA10_ORF22
*C. coli* 14983A (CP017025)	ATGGAGAAGAGATAAATAAACAAGTTTTAAGC (32/32)	DA10_ORF23
*C. coli* BP3183 (CP017871)	TCTTATAAACAAAGCGGTAATGAAGGCTTA (30/30)	DA10_ORF24
*C. jejuni* GB1 (HQ378304)	TGGTATGAGTTAGCAACTTATAATAACTCTAATGTTATTA (37/40)	DA10_ORF25
*C. jejuni* F280 (EF017337)	ATTTTGGAAGTACTGCTAGAAATGCAAGTTTTA (33/33)	DA10_ORF26
*C. coli* YH502 (CP018900)	TAGATAACGAGTTATATTCAGAAATACCAAA (31/31)	DA10_ORF27
*C. jejuni* FORC_046 (CP017229)	TGCAGAAAATGGAGATAGACAAGATTTTCCTGT (33/33)	DA10_ORF28
*C. jejuni* 183 (KR155181)	TCAAAGTGCTGAAAAAGATAGAAACTTACTTA (32/32)	DA10_ORF29
–	No matches	DA10_ORF30
*C. jejuni* HCC60 (KR155155)	CCTTATAAAAGCGAATATTTCACTGCGTGCG-TTATAC (36/38)	DA10_ORF31
*C. jejuni* NCTC 12662 (CP019965)	CCATCAAGTCGTGCAATTTTAATACACTGGG (30/31)	DA10_ORF32
*C. jejuni* NCTC 11168 (LS483362)	AGTTAAATTCAACTAATGAAACTTTGGAAAA (31/31)	DA10_ORF33
*C. jejuni* CJ066CC508 (CP0122224)	GTTATACTTACACCCTTTAAAAACTAATGG (30/30)	DA10_ORF34
*C. jejuni* YH003 (CP041584)	CTTTATGTAATTTTTATAATCAAAGTATAA (30/30)	DA10_ORF35
*C. jejuni* GB1 (HQ378304)	TCTACAAGAATGAGGATGATGATATTTTACAA (32/32)	DA10_ORF36
–	No matches	DA10_ORF37
*C. jejuni* NCTC 12660 (CP028910)	AGATGCTTTAACAGTATGTTCTTTAGCGGG (29/30)	DA10_ORF38
*C. jejuni* HCC04 (KR155185)	CCAAAACAATAGCAAATTTTAAATATTATATA (32/32)	DA10_ORF39
*C. jejuni* R133 (HQ378258)	ATGAGTAAAAAAGATAGTCAAGAATGGT (28/28)	DA10_ORF40
*C. jejuni* F007 (EF017341)	AAAAGAGCCTTTAGAAAACAATGTTTTTATAATA (31/34)	DA10_ORF41
*C. coli* RM4661 (CP007181)	ATAATTCCTAATAAAAAAGTAAGTTTTGATG (31/31)	DA10_ORF42
*C. jejuni* F459 (EF017334)	AAA-TAGTTTATAAAAATAAGCAAGATGTAGA (31/32)	DA10_ORF43
*C. jejuni* F226 (EF017339)	TTATTATGATGATAACTTAAATAAGTTTATC (31/31)	DA10_ORF44
–	No matches	DA10_ORF45
–	No matches	DA10_ORF46
–	No matches	DA10_ORF47
–	No matches	DA10_ORF48
*C. jejuni* NCTC 12660 (CP028910)	TTTAAATTTCAAAGATGAGAATATAGCTAA (30/30)	DA10_ORF49
–	No matches	DA10_ORF50
–	No matches	DA10_ORF51
*C. jejuni* NCTC 13257 (LR134502)	TAAAATAATTTGCAAGGTATATAAAAAATTTGATTTTG (37/38)	DA10_ORF52
–	No matches	DA10_ORF53
–	No matches	DA10_ORF54
*C. jejuni* R15 (HQ378302)	TTAAAAATAGAAATTTTATAGGTTGCGAAA (30/30)	DA10_ORF55
*C. jejuni* 80 (KR155173)	AAA-AGAAACTATATTAGCAGGTATAAATAATCT (33/34)	DA10_ORF56
–	No matches	DA10_ORF57
*C. jejuni* NCTC 12664 (CP028912)	GTGGCTAAGAATAAAATAAGAAACACTGGT (29/30)	DA10_ORF58
–	No matches	DA10_ORF59

While ~ 75% of the coding regions of the DA10 genome are actively targeted by *Campylobacter* CRISPR systems it is apparent that the remainder of the ORFs are not represented as spacer sequences. Analysis of ORFs that avoid CRISPR-mediated responses indicates that protection from immunity may be afforded for several reasons. Genes that have host homologues (as determined by conservation across a wide number of *C. jejuni*/*C. coli* genomes) will not be targeted without a detrimental effect on the host, and thus are spared from spacer acquisition due to the deleterious effects of autoimmunity. ORFs that are observed to be conserved between DA10 and other *Campylobacter* phages also appear to be free from CRISPR-mediated responses. This is perhaps unsurprising because homologues encoded in *Campylobacter* phage genomes that have evolved to successfully infect and target *Campylobacter* spp. are unlikely to be CRISPR targets. Phage-mediated circumvention of Type II-C CRISPR-mediated immunity in *C. jejuni* is a documented phenomenon. Class II and Class III *Campylobacter* phages encode conserved Cas4-like proteins that drive acquisition of host-derived CRISPR spacers. Consequently, Class II and III *Campylobacter* phage genomic DNA remains protected from CRISPR-mediated immunity during the infection cycle [23, 25]. DA10 and DA10-like phages do not encode a CRISPR Cas4-like protein. It is plausible that the distinct lack of ability to control the *Campylobacter* host CRISPR immune response has driven this family of phages to near extinction within the global *Campylobacter* population. In relation to the integrated DA10-like prophage encoded in CJ677CC520, it is reported that genome sequences derived from members of this clade encode atypical CRISPR systems. Deletion of essential *cas* genes will lead to a loss of functionality of the type II-C systems for CJ677-types [32]. For CJ677CC520 that lacks *cas1*, the ability to generate protospacers during phage infection has likely been lost due to absence of the DNA endonuclease function provided by Cas1 protein. Analysis of a Type II-C CRISPR system in *Riemerella anatipestifer* shows that *cas1/cas2* gene knockouts lose the ability to acquire protospacers, with functionality of the system restored upon ectopic restoration of the *cas1*/*cas2* genes [37]. In the absence of a functional CRISPR-cas system CJ677-types can remain a refuge for the DA10 genome, and kernels from which the phage population can expand when susceptible hosts become available that do not carry DA10-directed CRISPR spacers. However, re-emergence of the phage will depend upon the host maintaining the integrity of the phage genome, which does not appear to be the case for CJ677CC520.

## Conclusions

Bacteriophage DA10 has the shortest genome of any *Campylobacter* phage DNA recorded to date and represents a replicative form of a prophage present in the genome of *C. jejuni* CJ677CC520. DA10-like prophage sequences show a restricted distribution in the host genomes of *C. jejuni* and *C. coli* available in the sequence read archive compared to previously recognised Mu-like and CJIE-like prophage sequences. DA10 and DA10-like bacteriophages are a target for CRISPR-mediated immunity, which may be a driving factor in their rarity since 75% of the ORFs of DA10 are present as ~ 30 bp spacer sequences in numerous *Campylobacter* Type II-C CRISPR arrays. Under these circumstances, prophage integration could render the host susceptible to CRISPR-mediated autoimmunity and elimination. CJ677CC520 is among a clade of campylobacters that exhibit atypical CRISPR systems that are deficient *cas* genes, and in the absence of CRISPR-mediated immunity permit prophage integration.

## Methods

### Host strains & bacteriophage DA10 propagation

*Campylobacter jejuni* was routinely grown on blood agar base No.2 plates (BA plates; Oxoid) supplemented with 5% horse blood (TCS Biosciences) under microaerobic conditions in either a modular atmosphere controlled cabinet (5% CO_2_, 5% O_2_, 2% H_2_, 88% N_2_) or in anaerobic jars using gas replacement (7.3% CO_2_, 5.6% O_2_, 3.6% H_2_, 83.5% N_2_) at 42 °C. Bacteriophage DA10 was isolated as described previously [26] and propagated by plate lysis of the host *C. jejuni* GM. *C. jejuni* GM cells were collected from a BA plate and suspended in 10 mM MgSO4 (Thermo Fisher) to a cell density of approximately 10^7^ CFU/ml. A volume of 500 μl of the cell suspension was added 10^7^ PFU DA10 before adding 5 ml of molten NZCYM top agar (0.6%, VWR Leicestershire, UK) and pouring on top of a NZCYM agar plate.

### Pulse fields gel electrophoresis

For PFGE, plugs were prepared as described previously [38]. Briefly a 10 μl suspension of bacteriophage at 10^10^ PFU/ml was added to 40 μl of TE buffer (10 mM Tris, 1 mM EDTA [pH 7.5]) and mixed with an equal volume of 1.4% molten low-melting point PFGE agarose in TE buffer and dispensed into plug molds (Bio-Rad). Solidified plugs were washed in 5 ml of lysis buffer (100 mM EDTA, 10 mM Tris [pH 7.2], 1% Sarkosyl [w/v], 0.5 mg of proteinase K; Thermofisher) at 55 °C for 18 h with gentle shaking. The lysis solution was discarded and proteinase K inactivated by washing in 5 ml of 1 mM phenylmethylsulfonyl fluoride 50 mM EDTA, 20 mM Tris [pH 7.2] for 1 h at room temperature, before three successive washes of 20 min in 50 mM EDTA, 20 mM Tris [pH 7.2]. Agarose gels (1% w/v) were loaded with 2 mm plug slices. The gel was run using a Bio-Rad CHEF DRII system in 0.5× TBE for 18 h at 6 V/cm with a switch time of 30 to 60 s. Lambda concatemers were used as markers (GelSyringe™ New England Biolabs, USA), and the DNA was visualized by staining with ethidium bromide.

### Transmission electron microscopy

Glutaraldehyde-fixed phage suspensions on Pioloform grids were negatively stained with 0.5% uranyl acetate. The specimens were imaged using a Tecnai G12 biotwin TEM, run at 100Kv, with a SIS megaview camera system and Gatan Microscopy Suite software (Gatan Inc).

### Bacteriophage genomic DNA preparation

Genomic DNA was extracted from the DA10 lysate using a modified protocol of the Wizard DNA clean up kit (Promega, Southampton, UK). Phage suspensions were mixed with nuclease solution (10 mg/ml DNase and RNase) and incubated at 37 °C for 30 min. A precipitant solution containing 10% PEG 8000, 1 M NaCl was added at a ratio of 1:2 precipitant to lysate. The solution was mixed by inversion and incubated at 4 °C overnight followed by centrifugation at 10,000 g at 4 °C for 10 min. The supernatant was discarded, and the pellet resuspended in 500 μl of 5 mM MgSO4 and cleared by centrifugation for 10 s in a microfuge. Residual nucleases were degraded by addition of 10 μl of 0.5 M EDTA pH 8 and proteinase K to a final concentration of 0.1 mg/ml. The purification resin supplied with the Promega Wizard kit was resuspended and 1 ml added to the phage suspension. The mixture was inverted 5 to 6 times and filled into a 3 ml syringe (Part# A809B). A Wizard minicolumn was placed into a reaction tube and the syringe barrel attached to the column and the resin/lysate mix was pushed into the column until all liquid was forced through the resin. The column was washed with 2 ml of 80% isopropanol and centrifuged at 13,000 g for 2 min to dry the resin. The column was placed in a 1.5 ml microcentrifuge tube and 100 μl of sterile water at 80 °C pipetted into the column. The column was centrifuged at 13.000 g for 1 min to elute the DNA. Residual guanidine contaminants were removed by ethanol precipitation.

### Genome sequencing

Library preparation of DA10 genomic DNA followed the Illumina Nextera™ tagmentation protocol (Illumina, Cambridge, UK). The DA10 genome was assembled de novo using CLC Genomics Workbench Version 9.0.1 (Qiagen Bioinformatics, Aarhus, Denmark) from 2.1 million Illumina MiSeq paired-end sequence reads of 120 to 240 nucleotides.

### DA10 genome analysis

The DA10 nucleotide sequence was initially annotated using a combination of the online tools RAST (http://rast.nmpdr.org/rast.cgi) [39], Phaster (http://phaster.ca/) [40], and HHpred (https://toolkit.tuebingen.mpg.de/tools/hhpred) [41]. Manual curation of DA10 ORFs was performed using a combination of BLASTn and BLASTp to assist in annotation and assigning function to genes when possible [42]. The annotated DA10 DNA sequence was submitted to NCBI (Accession No. MN530981). Average nucleotide identities of DA10 and DA10-like phages were calculated from whole genome alignments using a pairwise BLAST comparative approach, and MUMMER was utilized for coding region alignments (https://github.com/mummer4/mummer). Analysis of DA10 ORFs represented as spacers present in *Campylobacter* spp. CRISPR arrays was performed using a combination of BLASTn and CRISPR-Cas++ (https://crisprcas.i2bc.paris-saclay.fr/) [43, 44]. Protospacer adjacent motif (PAM) sequence alignments were obtained using WebLogo 3 (http://weblogo.threeplusone.com/) [45, 46].

## Data Availability

All data generated or analysed during this study are included in this published article. DNA sequences appear in the National Center for Biotechnology Information (NCBI) nucleotide sequence database accession number MN530981. The bacteriophage is available from DA.

## Abbreviations

ANI: Average nucleotide identity
BLAST: Basic Local Alignment Search Tool
Cas: CRISPR-associated protein
CRISPR: Clustered Regularly interspaced Short Palindromic Repeats
CJIE: *Campylobacter jejuni* integrated elements
ORF: Open Reading Frame
PAM: Protospacer Adjacent Motif
PHASTER: Phage Search Tool Enhanced Release
RAST: Rapid Annotation using Subsystem Technology
